# Clinical Benefits of Sodium–Glucose Cotransporter 2 Inhibitors and the Mechanisms Underlying Their Cardiovascular Effects

**DOI:** 10.1016/j.jacasi.2022.03.009

**Published:** 2022-06-07

**Authors:** Yoshiaki Kubota, Wataru Shimizu

**Affiliations:** Department of Cardiovascular Medicine, Nippon Medical School, Tokyo, Japan

**Keywords:** diabetes, heart failure, sodium–glucose cotransporter 2 inhibitor, ATP, adenosine triphosphate, DPP-4, dipeptidyl peptidase-4, HF, heart failure, HFpEF, HF with preserved ejection fraction, HFrEF, HF with reduced ejection fraction, SGLT2, sodium–glucose cotransporter 2, T2DM, type 2 diabetes mellitus

## Abstract

In addition to showing antidiabetic effects, sodium–glucose cotransporter 2 (SGLT2) inhibitors also reduce cardiovascular events in patients with type 2 diabetes mellitus. In major trials of cardiovascular outcomes, SGLT2 inhibitors have been shown to improve cardiovascular and renal outcomes, including reduced rehospitalization in patients with heart failure, regardless of the presence of diabetes. A recent report showed that the benefits of SGLT2 inhibitors in terms of cardiovascular deaths/admissions caused by heart failure and reduced ejection fraction were greater in Asians than in Whites. In this review, the first part demonstrates the results of recent clinical trials and their clinical implications and outlines current trials and upcoming research areas. The second part provides a general overview of the current understanding of the mechanisms of the cardiovascular benefits of SGLT2 inhibitors.

## Highlights

• Type 2 diabetes mellitus and heart failure are closely related.
• SGLT2 inhibitors can reduce the incidence of cardiovascular events and heart failure.
• SGLT2 inhibitors can improve hemodynamics, myocardial energy supply, and sympathetic and parasympathetic nerve activities.

The incidence of heart failure (HF) is increasing worldwide.^1^ Type 2 diabetes mellitus (T2DM) and HF are closely related to each other,^2^, ^3^, ^4^ and approximately 45% of people with HF have diabetes.^5^ The risk of HF is correlated with hemoglobin A1c levels.^6^^,^^7^ HF is the most common cause of hospitalization caused by cardiovascular events in patients with diabetes.^8^ T2DM and HF have independent prognoses.^9^, ^10^, ^11^ In Asia, an aging population and a significant increase in cardiovascular risk factors have affected the HF burden.^12^ Asian patients with HF and a reduced ejection fraction (HFrEF) show clinical features different from other patients.^13^^,^^14^ The ASIAN-HF (Asian Sudden Cardiac Death in Heart Failure) registry was established to address the lack of knowledge of the burden associated with chronic HF among Asian patients.^15^ In this prospective observational cohort, the comorbidities of diabetes had the largest influence on the combined outcome of death and hospital admission caused by HF.^16^ Based on evaluations using the ASIAN-HF registry, Asian patients with HF had a lower mean body mass index and were less likely to be in New York Heart Association functional class III or take angiotensin-converting enzyme inhibitors.^17^

The high prevalence of T2DM in Asia has the potential to cause an outbreak of cardiovascular diseases and HF, so prevention of cardiovascular complications is essential in managing patients with T2DM.^18^^,^^19^ The current prevalence rates of T2DM and cardiovascular diseases and estimates of future risk in Asia are alarming and require effective action to prevent disease development and ensure effective management of T2DM and cardiovascular diseases. In recent large randomized placebo-controlled trials, sodium–glucose cotransporter 2 (SGLT2) inhibitors were shown to decrease cardiovascular events, specifically secondary prevention and hospital admission for HF.^20^, ^21^, ^22^, ^23^, ^24^, ^25^, ^26^ Recent reviews have focused on the clinical benefits and mechanisms of the cardiorenal effects of SGLT2 inhibitors.^27^, ^28^, ^29^ In this review, the first part demonstrates the results of recent clinical trials and their clinical implications and outlines current trials and upcoming research areas. The second part provides a general overview of the current understanding of the mechanisms of the cardiovascular benefits of SGLT2 inhibitors. As a unique point, we show on diabetes and HF as well as SGLT2 inhibitors in the Asian population.

## SGLT2 Inhibitors and HFrEF

In 4 cardiovascular outcome trials (ie, EMPA-REG OUTCOME [Empagliflozin Cardiovascular Outcome Event Trial in Type 2 Diabetes Mellitus Patients], CANVAS [Canagliflozin Cardiovascular Assessment Study], DECLARE-TIMI 58 [Multicenter Trial to Evaluate the Effect of Dapagliflozin on the Incidence of Cardiovascular Events-Thrombolysis In Myocardial Infarction 58], and CREDENCE [Evaluation of the Effects of Canagliflozin on Renal and Cardiovascular Outcomes in Participants With Diabetic Nephropathy])^20^, ^21^, ^22^, ^23^ (Table 1) and 3 HF-specific trials (ie, DAPA-HF [Dapagliflozin and Prevention of Adverse-Outcomes in Heart Failure], EMPEROR-Reduced [Empagliflozin Outcome Trial in Patients With Chronic Heart Failure With Reduced Ejection Fraction], and SOLOIST-WHF [Effect of Sotagliflozin on Cardiovascular Events in Patients With Type 2 Diabetes Post Worsening Heart Failure)^25^^,^^26^^,^^30^ (Table 2), SGLT2 inhibitors significantly decreased the risk of all-cause mortality, cardiovascular mortality, and hospitalization caused by HF in both the presence and absence of T2DM. A trend toward reduced HF hospitalization-related outcomes was observed in the subgroup of patients with HF with preserved ejection fraction (HFpEF), but this conclusion was not definitive and should be considered exploratory. In comparison with placebo, SGLT2 inhibitors have not been shown to increase the risk of severe adverse events or discontinuation of treatment after adverse events. The 3 HF-specific trials also showed significant reductions in the composite outcome of initial hospitalization caused by HF or cardiovascular deaths. Although the DAPA-HF trial showed a significant reduction in mortality caused by cardiovascular and other causes, EMPEROR-Reduced and SOLOIST-WHF did not. A meta-analysis of these 3 trials showed a significant decrease in cardiovascular mortality from all causes.^31^ The results of a post hoc analysis of the results of cardiovascular trials further supported the benefits of reduced mortality in patients with HF.^31^ Subsequent analyses in the DAPA-HF trial reported that the benefits of dapagliflozin were significant in both patients with and without diabetes.^32^ Several other ongoing trials have focused on the role of SGLT2 inhibitors in patients with HFpEF, with or without T2DM. The mechanism underlying the benefits of SGLT2 inhibitors for the prognosis of patients with HFrEF is unclear, but some studies have suggested that they include beneficial effects on myocardial metabolism, fibrosis, inflammation, and vascular function.^33^, ^34^, ^35^

**Table 1:** 4 Cardiovascular Outcome Trials Involving SGLT2 Inhibitors

	EMPA-REG OUTCOME^20^	CANVAS Program^21^	DECLARE-TIMI 58^22^	CREDENCE^23^
SGLT2 inhibitor	Empagliflozin	Canagliflozin	Dapagliflozin	Canagliflozin
Median duration of follow-up, y	3.1	2.4	4.2	2.6
All	7,020	10,142	17,160	4,401
Asian	1,517 (21.6)	1,248 (12.7)	2,303 (13.4)	877 (19.9)
MACE	HR: 0.86 (95% CI: 0.74-0.99); *P =* 0.04	HR: 0.86 (95% CI: 0.75-0.97); *P =* 0.02	HR: 0.93 (95% CI: 0.84-1.03); *P =* 0.17	HR: 0.80 (95% CI: 0.67-0.95); *P =* 0.01
CV death	HR: 0.62 (95% CI: 0.49-0.77); *P <* 0.001	HR: 0.87 (95% CI: 0.75-0.97)	HR: 0.83 (95% CI: 0.73-0.95); *P =* 0.005	HR: 0.78 (95% CI: 0.61-1.00); *P =* 0.05

Values are n or n (%) unless otherwise indicated.

CANVAS = Canagliflozin Cardiovascular Assessment Study; CREDENCE = Evaluation of the Effects of Canagliflozin on Renal and Cardiovascular Outcomes in Participants With Diabetic Nephropathy; CV death = cardiovascular death; DECLARE-TIMI 58 = Multicenter Trial to Evaluate the Effect of Dapagliflozin on the Incidence of Cardiovascular Events-Thrombolysis In Myocardial Infarction 58; EMPA-REG OUTCOME = Empagliflozin Cardiovascular Outcome Event Trial in Type 2 Diabetes Mellitus Patients; MACE = major adverse cardiovascular events including a composite of death from cardiovascular causes; SGLT2 = sodium–glucose cotransporter 2.

**Table 2:** 3 HF-Specific Trials Involving SGLT2 Inhibitors

	DAPA HF^25^	EMPEROR-Reduced^26^	SOLOIST-WHF^30^
SGLT2 inhibitor	Dapagliflozin	Empagliflozin	Sotagliflozin
Median duration of follow-up, m	18.2	16	9
All	4,744	3,730	1,222
Asian	1,076 (22.7)	493 (13.2)	15 (1.2)
Primary outcome^a^	HR: 0.74 (95% CI: 0.65-0.85); *P <* 0.001	HR: 0.75 (95% CI: 0.65-0.86); *P <* 0.001	HR: 0.67 (95% CI: 0.52-0.85); *P <* 0.001
CV death	HR: 0.82 (95% CI: 0.69-0.98)	HR: 0.92 (95% CI: 0.75-1.12)	HR: 0.84 (95% CI: 0.58-1.22); *P =* 0.36

Values are n or n (%) unless otherwise indicated.

DAPA-HF = Dapagliflozin and Prevention of Adverse-Outcomes in Heart Failure; EMPEROR-Reduced = Empagliflozin Outcome Trial in Patients With Chronic Heart Failure With Reduced Ejection Fraction; SOLOIST – WHF = Effect of Sotagliflozin on Cardiovascular Events in Patients With Type 2 Diabetes Post Worsening Heart Failure; other abbreviations as in Table 1.

a Composite of worsening heart failure (hospitalization or an urgent visit resulting in intravenous therapy for heart failure) or cardiovascular death.

### SGLT2 inhibitors and HFpEF

In contrast to the findings for HFrEF, the effects of SGLT2 inhibitors on patients with HFpEF are still limited. Data from a pooled analysis including the DECLARE-TIMI 58 trial, the SOLOIST-WHF, and SCORED (Effect of Sotagliflozin on Cardiovascular and Renal Events in Patients With Type 2 Diabetes and Moderate Renal Impairment Who Are at Cardiovascular Risk) trials showed borderline significant reduction in the composite outcome of HF hospitalization or cardiovascular death.^31^ EMPEROR-PRESERVED (Enpagliflozin Outcome Trial in Patients With Chronic Heart Failure With Preserved Ejection Fraction), in which 11% of the patients were Asians, enrolled 5,988 patients with HFpEF with and without T2DM. The primary outcome was hospitalization for HF or cardiovascular mortality. The results indicated that empagliflozin reduced HF and cardiovascular mortality.^36^ The DELIVER (Dapagliflozin Evaluation to Improve the Lives of Patients with Preserved Ejection Fraction Heart Failure; NCT01297257) trial is ongoing.

### SGLT2 inhibitors in asia

In a pooled analysis, empagliflozin was shown to be well-tolerated by East Asian patients with T2DM considering an exposure of more than 2,100 patient-years, consistent with the results for the overall population tested.^37^ Empagliflozin has also been shown to reduce the risk of cardiovascular outcomes and mortality not only in the overall study population but also in Asian patients with T2DM and a history of cardiovascular disease.^38^ In a large, international study involving patients with T2DM from the Asia Pacific, the Middle East, and North America, the use of SGLT2 inhibitors decreased cardiovascular events in a broad evaluation of patient outcomes and characteristics.^39^ Empagliflozin treatment is also associated with a lower risk of HF, all-cause mortality, and end-stage renal disease in comparison with dipeptidyl peptidase-4 (DPP-4) inhibitors in routine clinical practice in Japan, South Korea, and Taiwan.^40^ One study suggested that the use of SGLT2 inhibitors afforded cardiovascular disease protection and could be used safely in older adults with T2DM.^41^ In the meta-analysis of Asians, the effects of empagliflozin and dapagliflozin on hospitalization caused by HF were shown to be similar in 2 independent trials, suggesting that these drugs improve renal outcomes and reduce all-cause and cardiovascular mortality in patients with HFrEF.^42^ Similarly, in Asia, SGLT2 inhibitor treatment is an evidence-based therapeutic regimen for primary prevention of hospitalizations caused by HF and secondary prevention of cardiovascular events in patients with T2DM.^18^ Thus, SGLT2 inhibitors should be considered for additional dosing early in patients with multiple risk factors or pre-existing cardiovascular disease. A recent meta-analysis has shown that the benefits of SGLT2 inhibitors in Asians were greater than in Whites in terms of cardiovascular deaths/admissions caused by HF in patients with HFrEF.^43^

### Comparison with other antidiabetic drugs

Dapagliflozin has been reported to reduce the risk of HF and direct medical costs in comparison with DPP-4 initiators in Asian countries.^44^ Additionally, SGLT2 inhibitors have been shown to reduce the risk of cardiovascular events in comparison with initiators of other hypoglycemic agents and DPP-4 initiators in Japanese real-world practice.^45^ Although both SGLT2 inhibitors and glucagon-like peptide-1 receptor agonists were reported to reduce all-cause mortality, cardiovascular mortality, nonfatal myocardial infarction, and renal failure, SGLT2 inhibitors reduced mortality and hospitalization caused by HF more frequently than glucagon-like peptide-1 receptor agonists.^46^

### Mechanism of the HF improvements associated with SGLT2 inhibitors

**Figure 1:**
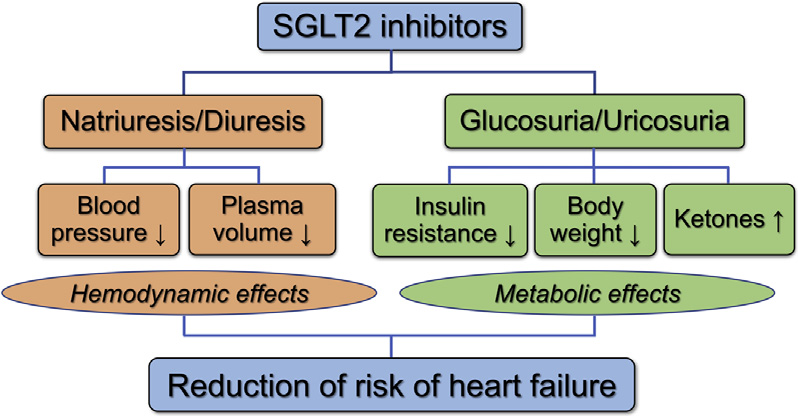
Main Mechanisms of Action of SGLT2 Inhibitors Their metabolic and hemodynamic effects improve myocardial function and reduce the risk of heart failure (details are provided in the text).^47^ SGLT2 = sodium–glucose cotransporter 2.

The beneficial effects of SGLT2 inhibitors on hemodynamics, myocardial energy supply, and sympathetic and parasympathetic nerve activities are illustrated in Figure 1.^47^^,^^48^

### Hemodynamic effect

The combination of the natriuretic and osmotic effects of SGLT2 inhibitors reduces intracellular and extracellular volumes to the same extent.^49^^,^^50^ The sustained reduction in intravascular volume and blood pressure reduces preload and postoperative load of the heart,^51^, ^52^, ^53^ respectively, alleviating the cardiac workload, and improving left ventricular function.^54^ SGLT2 inhibitors reduce reflex sympathetic hyperactivity and affect other neurohormonal pathways that affect the heart by altering intravascular volume and blood pressure hemodynamics, but do not increase the heart rate.^55^^,^^56^ Several clinical studies have reported significant body weight reductions in patients treated with SGLT2 inhibitors.^57^^,^^58^

### Myocardial energy supply effect

SGLT2 inhibitors increase the circulatory rates of ketone bodies.^59^ Ketones are freely absorbed by myocardial cells and can be a more effective source of adenosine triphosphate (ATP) than fatty acids for the failing heart.^60^ Additionally, the rate of use of ketones is reduced during myocardial ischemia.^61^ An experimental study reported that the increased use of fatty acids, ketone bodies, and branched-chain amino acids with empagliflozin inhibited the reduction of ATP and increased myocardial ATP levels.^62^ The mechanism underlying these effects involves the activation of the signal transducer and transcription activator 3, which, therefore, has antioxidant and anti-inflammatory activities.^63^ Several experimental and human studies have shown the beneficial effects of SGLT2 inhibition on cardiac remodeling.^64^, ^65^, ^66^, ^67^, ^68^, ^69^, ^70^ In a basic experimental design of acute myocardial infarction, SGLT2 inhibitors preserved heart function and reduced the infarct size.^63^ Furthermore, SGLT2 inhibitors have been reported to increase erythropoietin, which has cardioprotective effects, and hemoglobin, which enhances the oxygen supply to the myocardium.^71^^,^^72^ Additionally, SGLT2 inhibitors have been hypothesized to directly inhibit sodium–hydrogen (Na^+^/H^+^) exchange in the myocardium, resulting in an increase in mitochondrial calcium levels, improvement in mitochondrial function, reduction of oxidative stress, and reduction of arrhythmias.^33^ All of these mechanisms strongly suggest that SGLT2 inhibitors have cardioprotective effects.

### Effect on sympathetic and parasympathetic nerve activities

In our EMBODY trial, a prospective randomized placebo-control trial in patients with acute myocardial infarction associated with T2DM, the cardiac sympathetic nerve activity was significantly decreased, and the parasympathetic nerve activity was significantly increased in only the empagliflozin group.^73^ The Central Illustration shows our proposed mechanism for the decrease of cardiac sympathetic nerve activity by SGLT2 inhibitors. There are considered to be 3 mechanisms to decrease the cardiac sympathetic nerve activity by SGLT2 inhibitors, involving a hemodynamic effect, a metabolic (myocardial energy supply) effect, and a hepatic vagus nerve-mediated effect. The decreases of both myocardium oxygen consumption (myocardial energy supply effect) and cardiac preload and afterload (hemodynamic effect)^74^ are easily expected to decrease the cardiac sympathetic nerve activity. The vagus nerve in the liver controls neuron activation in the rostral raphe pallidus, which promotes sympathetic activity in the heart and increases the heart rate.^75^ Administration of SGLT2 inhibitors can reduce the activity of the cardiac sympathetic nerve by reducing the activity of the cord and controlling the heart rate.^76^ The precise mechanism for the increase of cardiac parasympathetic nerve activity with SGLT2 inhibitors demonstrated by our EMBODY trial is unclear, which may be a secondary effect caused by the decrease of cardiac sympathetic nerve activity. In any case, both the decease of sympathetic nerve activity and the increase of parasympathetic nerve activity in the heart with SGLT2 inhibitors indicate that SGLT2 inhibitors have preventive effects on cardiac arrhythmias as well as exacerbation of HF.

**Central Illustration:**
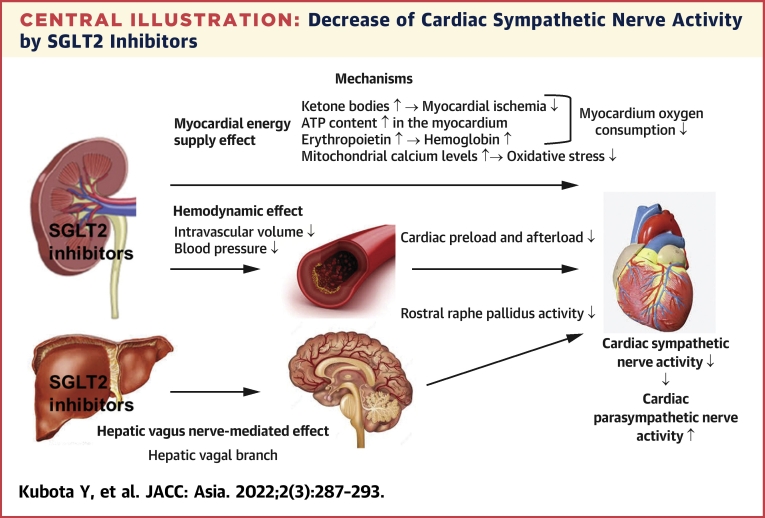
Decrease of Cardiac Sympathetic Nerve Activity by SGLT2 Inhibitors There are considered to be 3 mechanisms to decrease the cardiac sympathetic nerve activity by SGLT2 inhibitors, involving a hemodynamic effect, a metabolic (myocardial energy supply) effect, and a hepatic vagus nerve-mediated effect (details are provided in the text). ATP = adenosine triphosphate; SGLT2 = sodium–glucose cotransporter 2.

## Safety of SGLT2 Inhibitors

SGLT2 inhibitors may increase the risk of fungal genital infections, urinary tract infections, and euglycemic diabetic ketoacidosis.^77^^,^^78^ In the CANVAS trial, an increased risk of bone fractures and lower limb amputations was reported only with canagliflozin.^21^ Dosage adjustments are not required in older patients; the risk of adverse events related to volume depletion, renal failure, or urinary tract infection is higher in patients ≥65 years of age.^78^ The risk of euglycemic diabetic ketoacidosis is higher in lean patients, those with decreased β-cell reserves, and those on a ketogenic diet.^78^

## Conclusions

SGLT2 inhibitors can be used for the secondary prevention of cardiovascular outcomes in patients with T2DM and a history of cardiovascular disease in consideration of their beneficial cardiovascular and metabolic effects. SGLT2 inhibitors can also be used for primary and secondary prevention of HF-related hospitalization in patients with T2DM and multiple risk factors.

## Funding Support and Author Disclosures

Dr Shimizu has received honorariums and/or scholarship funds from Boehringer Ingelheim Co, Ltd, Daiichi Sankyo Co, Ltd, Ono Pharmaceutical Co, Ltd, Bayer Co, Ltd, Pfizer Co, Ltd, and Bristol-Myers Squibb Co, Ltd. All other authors have reported that they have no relationships relevant to the contents of this paper to disclose.
